# The statistical probability factor in triplet mediated photon upconversion: a case study with perylene†

**DOI:** 10.1039/d3tc03158f

**Published:** 2023-10-02

**Authors:** Lukas Naimovičius, Edvinas Radiunas, Manvydas Dapkevičius, Pankaj Bharmoria, Kasper Moth-Poulsen, Karolis Kazlauskas

**Affiliations:** a Institute of Materials Science of Barcelona, ICMAB-CSIC Bellaterra Barcelona 08193 Spain; b Institute of Photonics and Nanotechnology, Vilnius University Saulėtekio Av. 3 LT-10257 Vilnius Lithuania karolis.kazlauskas@ff.vu.lt; c Catalan Institution for Research & Advanced Studies, ICREA Pg. Lluís Companys 23 Barcelona Spain; d Department of Chemical Engineering, Universitat Politècnica de Catalunya, EEBE Eduard Maristany 10–14 08019 Barcelona Spain kasper.moth-poulsen@upc.edu; e Department of Chemistry and Chemical Engineering, Chalmers University of Technology Kemivagen 4 Gothenburg 412 96 Sweden

## Abstract

Triplet–triplet annihilation photon upconversion (TTA-UC) is a process where two low-energy photons are converted into one higher-energy photon. A crucial component for an efficient upconversion process is the statistical probability factor (*f*), defined as the probability of the formation of a high-energy singlet state upon coupling of two low-energy triplet states. Theoretically, *f* depends on the energy level distribution, molecular orientation, inter-triplet exchange coupling of triplet dyads, and spin-mixing of resulting spin states (singlet, triplet, and quintet). However, experimental values of *f* for acene-based annihilators have been subject to large variations due to many factors that have resulted in the reporting of different *f* values for the same molecule. In this work, we discuss these factors by studying perylene as a case study annihilator, for which by far the largest variation in *f* = 16 to 100% has been reported. We systematically investigated the TTA-UC of PdTPBP:perylene, as a sensitizer–annihilator pair and obtained the experimental *f* = 17.9 ± 2.1% for perylene in THF solution. This limits the maximum TTA-UC quantum yield to 9.0% (out of 50%) for this annihilator. We found that such a low *f* value for perylene is largely governed by the energy-gap law where higher non-radiative losses due to the small energy gap between 2 × T_1_ and T_2_ affect the probability of singlet formation. Interestingly, we found this observation true for other acene-based annihilators whose emission ranges from the UV to the yellow region, thus providing a blueprint for future design of efficient TTA-UC systems

## Abstract 2

Experimentally determined statistical probability factor in TTA-mediated photon upconversion for perylene is ∼18%, predominantly dictated by the energy-gap law. This limits the maximum TTA-UC quantum yield to 9.0 % (out of 50%) for this annihilator.

## Graphical abstract

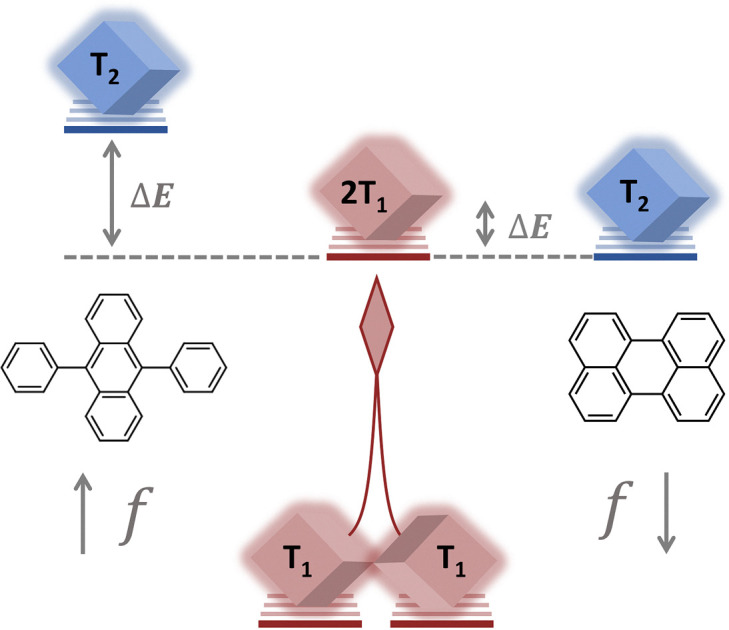

## Introduction

**Fig. 1:**
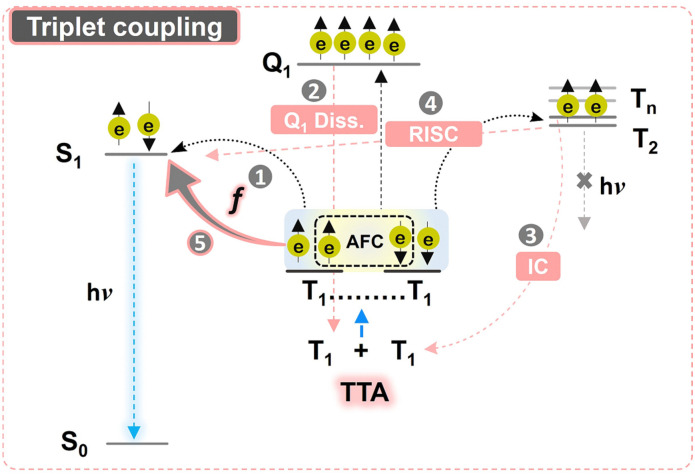
Schematic view of the processes contributing towards statistical probability factor *f* after triplet coupling post-TTA. The TTA results in the formation of a triplet exchange coupled pair (T_1_…T_1_) that can form 1 singlet-pair state (S_1_), 3 triplet-pair states (T_2_), and 5 quintet-pair states (Q_1_) in case of strong exchange coupling. The singlet-pair state contributes towards *f* directly *via* route 1, supported by Q_1_ dissociation (route 2). In case of weak exchange coupling, *f* can further be increased (route 5) if supported by internal conversion (IC, route 3) and reverse intersystem crossing (RISC, route 4).

Photon upconversion (UC) is a non-linear optical process that transforms low-energy photons into high-energy photons.^1^ Triplet–triplet annihilation photon upconversion (TTA-UC) is a sensitized UC process that converts two low-energy photons *via* triplet states to one high-energy photon emitted from the singlet state.^2,3^ TTA-UC is advantageous to other UC processes^1^ due to its ability to function upon incoherent low-density excitation at flexible spectral ranges.^4^ Hence, it is increasingly being applied in photovoltaics, bioimaging, photocatalysis, photodynamic therapy, sensing, optogenetics, and 3-D printing.^5–13^ A typical TTA-UC occurs in an ensemble of sensitizer and annihilator chromophores, where an excited triplet-state of the sensitizer after absorbing low energy photons sensitizes the annihilator triplets *via* Dexter energy transfer (DET), which then undergo TTA to produce a high *f* energy emissive singlet state. This process of the formation of a higher energy singlet state upon coupling of two low energy triplet states is described by the statistical probability factor (*f*) as shown in Fig. 1. However, there is a lot of ambiguity about the calculated *f* of various annihilators and their dependence on different photochemical and energetic parameters. In this work, we experimentally assess the *f* of the perylene annihilator and discuss the dependence of *f* on the energy gap law to optimize a suitable energetic design for annihilators with high *f* factor.

The strong exchange coupling of triplet states can result in nine possible triplet-pair spin eigen states with three spin multiplicities (1 singlet, 3 triplets, and 5 quintets) according to the Glebsch–Gordan series.^14^ Triplet coupling can be simply defined by Heisenberg's spin only Hamiltonian, eqn (1).^15,16^

1 *Ĥ* = −2*J*Ŝ_1_·Ŝ_2_

where Ŝ_1_ and Ŝ_2_ are individual spin operators of the two individual interacting triplets and *J* is the magnetic exchange parameter that also defines the strength of inter-triplet exchange interactions.

*J* contains all the spatial information of the wave function through-space and through-bond interactions which determine the ground-state spin preferences upon coupling. Due to anti-ferromagnetic coupling (AFC), the energetic order of spin multiplicities reverses against Hund's rule as per eqn (2) (Fig. 1).

2 *E*_s_ = −*J* (*S*(*S* + 1) − 4)

where *E*_s_ is the energy of the excited state and *S* is the total angular momentum of the coupling triplets.

Since the fate of eventual singlet emission after triplet coupling is decided by the *f* factor, it is imperative to understand its relevance with regard to calculations. One key ambiguity about *f* in TTA-UC is the obtained experimental values being higher than that of the spin-statistical limit of 1/9.^17–19^ This ambiguity is attributed to the dissociation of the quintet-pair state to individual triplets, reverse inter-system crossing (RISC) of the T_*n*_ state to the photo-emissive S_1_ state, and internal conversion of the triplet-pair state to individual triplets (Fig. 1).^20^ The quintet-pair state dissociates back to individual triplets because the quintet state is energetically inaccessible in a single chromophore.^21^ Hence, only 1 singlet-pair state and 3 triplet-pair states are directly involved in the spin statistics in the case of strong-exchange coupling. However, mixed spin states with singlet character can be formed in case of weak exchange coupling that can increase the *f* factor beyond 1/9.^20^

As discussed below, several other factors have a strong influence on the *f* value such as the chemical structure and energetics of a molecule. The chemical structure of a molecule can play an important role in determining the *f* value since it influences the molecular orientations (anisotropic interactions), Dexter distance,^22^ and transition dipole moment densities during inter-triplet exchange coupling.^23,24^ Hence, substitution can alter the triplet-pair wavefunction for an effective or ineffective coupling leading to a low or high *f* factor and *ϕ*_UC_.^17–19,23,24^

Besides these factors, the excited state energy level distribution is one of the most important factors since it has an impact on the energy transfer processes occurring within the system such as triplet recycling or losses to higher energy states *via* non-radiative decay. In the case of weak coupling, the rate of non-radiative decay (*k*_nr_) of the excited state can be understood from the energy gap law using eqn (3) and (4).^25,26^3
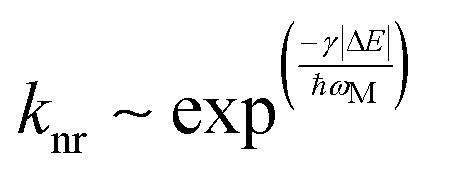
4
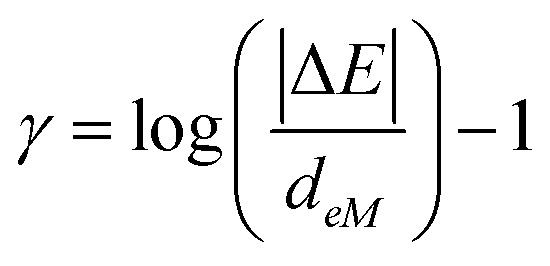
where *ħω* is a single vibrational frequency, *M* is the average displacement mode of the maximum frequency, and |Δ*E*| is the energy gap between electronic states. Hence, *k*_nr_ decreases exponentially with increasing Δ*E* and 1/*ħω*_M_.

For TTA to occur, the combined energy of the first two triplet states (2 × T_1_) of an annihilator is required to be higher than that of the first singlet state (*E*_2T1_ ≥ *E*_S1_). However, if the higher energy T_*n*_ states (T_2_, T_3_… *etc.*) are close enough to 2 × T_1_, it may lead to enhanced non-radiative losses that will hinder the generation of emissive S_1_. Therefore, the Δ*E*_2T1–S1_ and Δ*E*_2T1–T2_ energy gaps have a strong influence on *f*,^27–29^ which is discussed in this work.

**Fig. 2:**
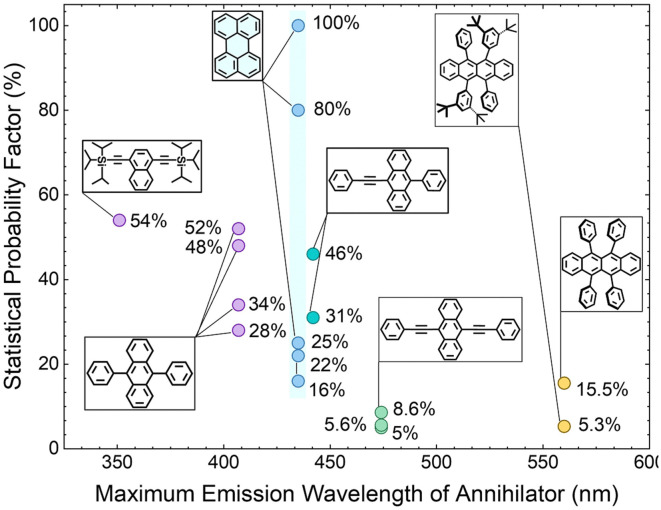
Variations in the reported experimental *f* values of various acene-based annihilators emitting from the UV to the yellow region of the spectrum (data with ref. no are provided in Table S2, ESI†). Asterisk (*) marks indicate the values determined by us from the available data as in the example represented in Table S3 and Fig. S5 (ESI†).

The variations in the reported *f* value are common for some acene-based annihilators emitting from the UV to the yellow region of the spectrum (Fig. 2 and Table S2, ESI†). In particular, large variations of *f* = 16–100% for perylene were reported in the literature.^30,31^ (Fig. 2, see Table S2 with ref., ESI†). This leads to *ϕ*_UC_ from low (*ϕ*_UC_ = 2–9%)^30,32–37^ to high values up to *ϕ*_UC_ = 38%^31^ in deaerated organic solution. *ϕ*_UC_ was reported to increase even further to 42% in dimerized perylene as an annihilator.^38^ Hence, we assess this difference of *f* by performing a systematic experimental and excited state modeling study to understand its relationship with the energy gap law.^25,26^

## Results and discussion

**Fig. 3:**
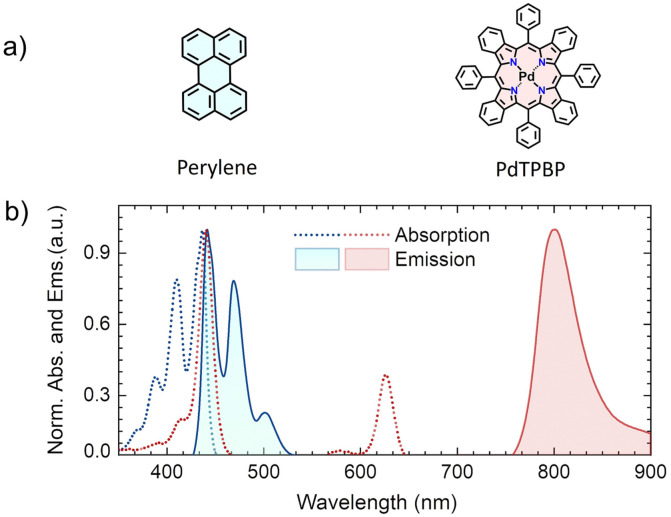
(a) Molecular structures of perylene (annihilator) and PdTPBP (sensitizer). (b) Absorption and emission spectra of perylene (20 μM), and PdTPBP (1 μM) in THF. Emission spectra were obtained upon excitation of perylene at 420 nm and PdTPBP at 640 nm CW lasers.

For experimental analysis, a systematic TTA study of perylene as an annihilator with palladium(ii) *meso*-tetraphenyl-tetrabenzoporphyrin (PdTPBP) as a sensitizer (ISC approaching unity^39^) is performed (Fig. 3a), to determine the key photophysical parameters and loss channels leading to the accurate calculation of the *f* value.

In general, *f* is calculated from eqn (5), considering the quantum yield of every single photophysical process within the system.5
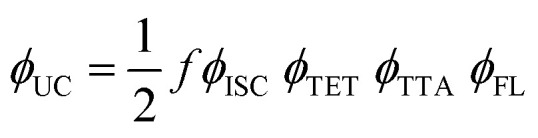
where *ϕ*_UC_, *ϕ*_ISC_, *ϕ*_TET_, *ϕ*_TTA_ and *ϕ*_FL_ are quantum yields of upconversion, intersystem crossing, triplet energy transfer from sensitizer to annihilator, triplet–triplet annihilation, and fluorescence of annihilator, respectively.

Due to the aggregation challenges of perylene above a certain concentration in organic solution (≈1 × 10^−3^ M),^32,34^ we carried out photophysical characterization and TTA-UC experiments at a concentration, where perylene shows only monomeric emission in tetrahydrofuran (THF). The experimental absorption and emission spectrum of perylene and PdTPBP are shown in Fig. 3b. The absorption maximum of perylene was observed at 436 nm (S_0_–S_1_) with accompanying vibronic bands at 410 and 388 nm, whereas the monomeric emission maximum was observed at 441 nm along with the shoulder peaks at 469 and 502 nm. The absorption spectrum of PdTPBP showed a characteristic Soret band at 440 nm and a Q-band at 626 nm, while the phosphorescence emission maximum was observed at 801 nm (1.55 eV). Due to the overlap of the absorption/emission spectra, perylene is prone to strong reabsorption effects at high concentrations that can affect the *ϕ*_UC_ and eventually the calculated *f*. Additionally, the overlap of its emission spectrum with the PdTPBP Soret absorption band can affect *ϕ*_UC_ due to back energy transfer *via* Förster resonance energy transfer (FRET).

**Fig. 4:**
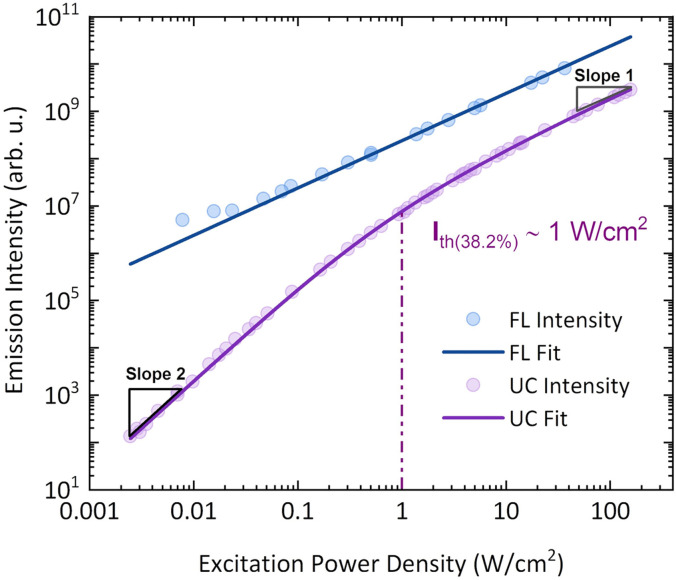
Linear dependence of perylene fluorescence emission (blue circles, *λ*_ex_ = 420 nm CW laser) and quadratic/linear dependence of perylene UC emission (violet circles, *λ*_ex_ = 640 nm CW laser) on the excitation power density in the UC solution. The *I*_th_ value was obtained by fitting the UC emission intensity using eqn (6).

For TTA-UC measurements, perylene:PdTPBP UC solution was prepared at 1 × 10^−4^ M and 1 × 10^−5^ M concentrations, respectively. The selected concentrations were reported^31,34^ to be optimal for TTA-UC investigation of perylene:PdTPBP solution. At the studied concentration of perylene in THF (1 × 10^−4^ M), a *ϕ*_FL_ = 96% ± 5% was determined without PdTPBP. The deaerated TTA-UC solution showed a UC emission maximum at 446 nm upon 640 nm laser excitation with *ϕ*_UC_ = 3.9% at ∼100 W cm^−2^ of excitation density (Fig. S1, ESI†). *ϕ*_UC_ was evaluated using two methods: the absolute quantum yield method, which utilizes an integrating sphere and the widely used relative method. The latter method tends to be more sensitive to the measurement configuration and reference standard. However, both methods produced consistent results, yielding a similar *ϕ*_UC_ of 3.9% (see Fig. S2 and Table S1, ESI†). The UC emission spectrum in our system is red-shifted by 0.04 eV compared to the emission maximum of the fluorescence spectrum without sensitizer. This is consistent with the decrease in *ϕ*_FL_ from 96% to 50% due to the reabsorption of the original emission maximum by the Soret band of the sensitizer *via* FRET (Fig. S3, ESI†). The maximum UC quantum yield (*ϕ*^∞^_UC_) needed for the determination of *f* was obtained only at high *I*_ex_ values where *ϕ*_TTA_ approaches unity according to eqn (6) proposed by Murakami *et al.*^40^ using threshold excitation intensity (*I*_th_) data (Fig. 4).6
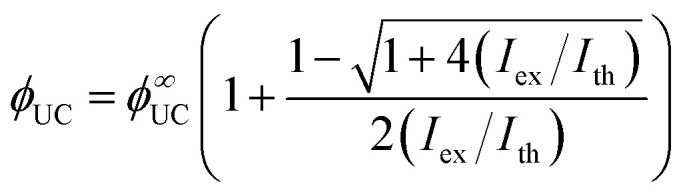
The quadratic/linear dependence of UC emission and the linear dependence of perylene fluorescence were obtained by the excitation of the UC solution at 640 nm and 420 nm, respectively (Fig. 4). To accurately evaluate *ϕ*^∞^_UC_ and *I*_th_ values, the laser excitation spot sizes for 420 nm and 640 nm lasers were recorded before each measurement (Fig. S4). Using eqn (6), *ϕ*^∞^_UC_ = 4.1% and *I*_th_ ∼ 1 W cm^−2^ were obtained. The obtained *ϕ*^∞^_UC_ value is close to that reported by the majority of research groups (Table S4, ESI†).

Further, to reliably estimate *ϕ*_TET_, three independent methods were employed, which are based on (1) the quenching of the sensitizer's phosphorescence lifetime (*τ*_P_), (2) the quenching of the sensitizer's quantum yield (*ϕ*_P_) by the annihilator, and (3) the rise-time of delayed fluorescence transients due to upconversion (*τ*_rise_).

**Fig. 5:**
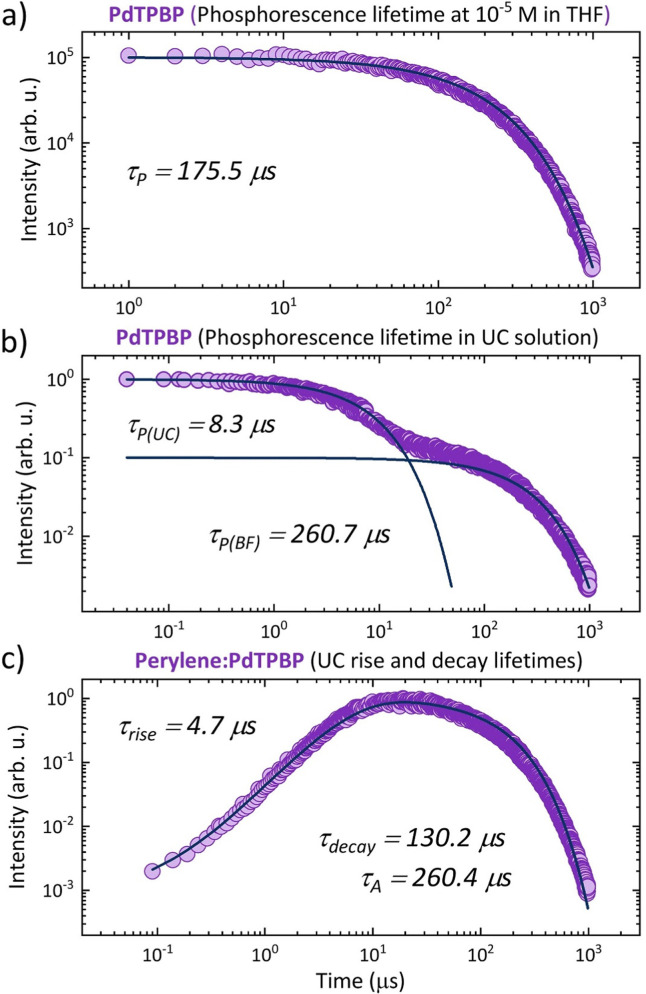
Transient rise or decay profiles of PdTPBP and perylene in deaerated THF solution. (a) Phosphorescence decay profile of PdTPBP in deaerated THF (1 × 10^−5^ M, *λ*_em_ = 800 nm); (b) Phosphorescence decay profile of PdTPBP in the TTA-UC solution (*λ*_em_ = 800 nm), and (c) rise and decay profile of the perylene UC emission in the TTA-UC solution (*λ*_em_ = 446 nm). All sample solutions were excited with a 640 nm laser at a 1 kHz repetition frequency. The blue lines indicate exponential fits of the rise or decay profiles according to the method reported elsewhere.^42^

From the phosphorescence transients of PdTPBP, the intrinsic *τ*_P_ was determined to be 175.5 μs (Fig. 5a), which is in accordance with the values reported elsewhere.^35,36^

In the TTA-UC solution, the phosphorescence of PdTPBP showed fast and slow decay components (Fig. 5b). The fast component showed a quenched phosphorescence lifetime (*τ*_P(UC)_) of 8.3 μs compared to 175.5 μs observed without an annihilator. This is due to the triplet energy transfer (TET) to perylene, resulting in *ϕ*_TET_ ∼ 95.2% according to eqn (7).7
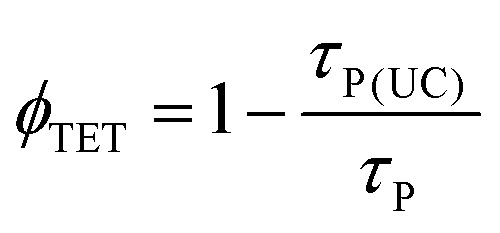
The slow decay component with a lifetime of 260.7 μs can be attributed to the back-FRET of upconverted singlets to the sensitizer (Fig. 3b) since it has a similar decay time to that of perylene's triplet state lifetime *τ*_A_ = 2 × *τ*_decay_ = 260.4 μs (Fig. 5c).^32,41^ The *ϕ*_TET_ was also determined from delayed fluorescence transients resulting from TTA.

The UC rise time (*τ*_rise_) was determined to be ∼4.7 μs (Fig. 5c), therefore leading to *ϕ*_TET_ ∼ 94.6% following eqn (8).8
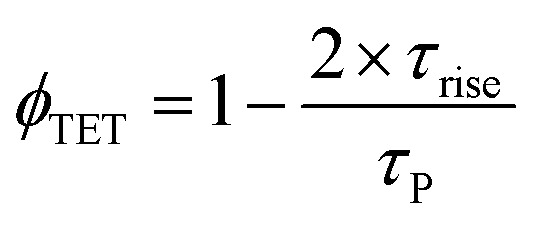
A high *ϕ*_TET_ = 88.2% was also confirmed by the decrease in phosphorescence quantum yield of PdTPBP (*ϕ*_P_ = 8.5%) without perylene to *ϕ*_P(UC)_ = 1% in the UC solution as per eqn (9).9
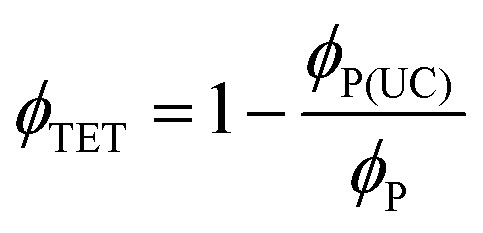
Finally, the average *ϕ*_TET_ from three methods was calculated to be 91.7% ± 3.5% which we use for the estimation of *f* value in this work.

All the determined photophysical parameters that lead to the eventual calculation of the *f* factor of perylene according to eqn (5) are tabulated in Table 1.

**Table 1:** Calculated values of the photophysical parameters of the PdTPBP:perylene UC system in deaerated THF

*ϕ*_ISC_	*ϕ*_TET_ a	*ϕ*_TTA_	*ϕ*_FL_	*ϕ* ^∞^_UC_	*f*
100%	91.7 ± 3.5%	100%	50 ± 5%	4.1 ± 0.2%	17.9 ± 2.1%

a *ϕ*_TET_ is provided as the average result of three independent methods.

To accurately determine the *f* and avoid any ambiguities regarding the excitation density, we used *ϕ*^∞^_UC_ when the *ϕ*_TTA_ approaches unity, instead of using *ϕ*_UC_. As follows, we calculated *f* = 17.9% ± 2.1% for perylene according to eqn (5), which is a 5-fold lower value than the one reported elsewhere^31^ at the same annihilator and sensitizer concentrations. Therefore, our calculated *f* value limits the maximal attainable UC yield (*ϕ*^∞^_UC_) of perylene-based TTA-UC systems to 
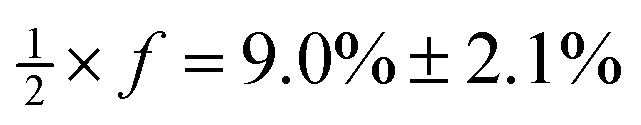
, which is 4-fold lower as compared to 38 ± 3%^31^ but in quantitative agreement with other reports^30,32–37^ (Table S4, ESI†).

**Fig. 6:**
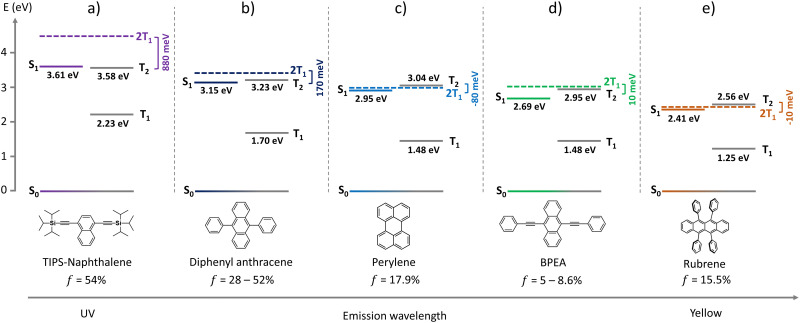
Calculated energy states of different acene-based annihilator chromophores emitting from the UV to the yellow region of solar spectrum. (a) TIPS-naphthalene; (b) diphenyl anthracene; (c) perylene; (d) BPEA; (e) rubrene. For comparison with experimental values see Fig. S6 and Table S5 (ESI†).

To better understand the possible loss or augmentation channel of *f* during triplet-coupling in perylene, we explored its relationship with the energy gap law.^25^ For that, we optimized the molecular geometry and calculated the excited state energies for different acene-based chromophores from the UV to the yellow region using density functional theory (Fig. 6) at the B3LYP/6-31(d)^43–45^ level. For perylene, the calculated first singlet state energy (S_1_ = 2.95 eV) is in accordance with the 0–0 absorption peak whereas the first triplet state (T_1_) was found to be at 1.48 eV and hence 2 × T_1_ is at 2.96 eV. The positive Δ*E*_2T1–S1_ = 10 meV supports the feasibility of TTA.^46,47^ Since 2 × T_1_ to T_2_ non-radiative decay is a possible loss channel for *f*, we analyzed it for perylene in comparison to other acene chromophores. In the case of perylene, we calculated T_2_ at 3.04 eV that leads to a small negative Δ*E*_2T1–T2_ = −80 meV, which is achievable with thermal energy at room temperature (*k*_B_*T*).^48–50^ Hence, it could be the possible non-radiative loss channel that negatively impacts *f* (Fig. 6c). Comparing it with those of TIPS-naphthalene (*f* = 54%) and DPA (*f* = 28–52%), high Δ*E*_2T1–T2_ values of 880 meV and 170 meV, respectively, were obtained (Fig. 6a and b). Such a high Δ*E*_2T1–T2_ in these cases seems to be the reason of lower *k*_nr_ and hence higher *f* factor. A similar conclusion can be drawn from eqn (3), for the lower *f* factor of BPEA (*f* = 5 – 8.6%), and rubrene (*f* = 15.5%) having Δ*E*_2T1–T2_ = 10 meV and Δ*E*_2T1–T2_ = −10 meV (Fig. 6d and e), respectively, which supports a higher *k*_nr_ due to the low Δ*E*, achievable with *k*_B_*T*. Hence, the energy gap law is indeed one of the key factors contributing to the low *f* value of perylene, BPEA, and rubrene due to non-radiative loss. Similarly, recently reported^51^ novel diketopyrrolopyrrole-based annihilators exhibit the same pattern when decreasing Δ*E*_2T1–T2_ hinders the *f*, thus pointing to the dependence on the energy gap law and hence generalizing this notion.

## Conclusions

In summary, we performed a systematic study of a perylene:PdTPBP upconversion system and quantitatively evaluated all energy transfer processes occurring within this system for reliable determination of the statistical probability factor (*f* = 17.9% ± 2.1%) of perylene in solution. The low *f* value sets the upper limit of *ϕ*_UC_ for perylene-based TTA-UC systems to 
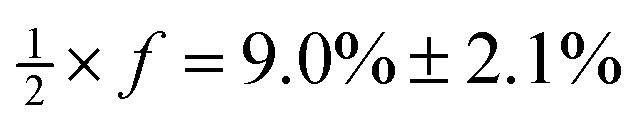
. We note that the erroneous evaluation of the *f* factor occasionally happens, which in the case of perylene has led to a reported 5-fold higher value suggesting it to be an ideal TTA annihilator. However, this can be attributed to inaccurate determination of other contributing processes, subsequently resulting in the large variation of the *f* value for the same molecule. We find that the low *f* value of perylene originates from higher non-radiative losses of the 2 × T_1_ to T_2_ states due to their proximity (the energy-gap law). Interestingly, we also discover a generalized trend of decreasing *f* for acene-based annihilators emitting from the UV to the yellow region, that is governed by this law. This confirms that the energy-level distribution of different annihilators is the prime reason for their diverse *f* factor and emphasizes the relevance of the energy gap law in understanding the probability of singlet state formation *via* TTA. Finally, the work provides a blueprint to design future annihilator molecules with a higher *f* factor for practical applications.

## Experimental

### Materials

Tetrahydrofuran (THF) solvent, perylene, and PdTPBP were purchased from Sigma-Aldrich.

### Preparation of solutions

All solutions studied were prepared using the THF solvent. The perylene compound was dissolved to produce a concentrated stock solution of 1 mM, while the sensitizer PdTPBP solution of 0.1 mM was prepared. The concentrated emitter stock solution of 1 mM was diluted to 2 × 10^−5^ M to perform photophysical measurements. Further on, the TTA-UC solution containing 10^−4^ M of perylene and 10^−5^ M of PdTPBP was prepared. All the samples were prepared in a glovebox under a nitrogen atmosphere with O_2_ and H_2_O concentrations <0.1 ppm. The two-millimeter width cuvette with a screw cap containing the investigated solution was sealed. This ensured necessary protection from triplet quenching by reactive oxygen species.^52^

### Optical techniques

The absorption spectra of the samples were recorded using a spectrophotometer FLS980 (Edinburgh Instruments). For sample excitation at 420 nm (30 mW) or 640 nm (50 mW), continuous-wave semiconductor lasers (Picoquant) were used. The steady-state FL and UC emission spectra were measured using a back-thinned CCD spectrometer PMA-12 (Hamamatsu). The long-lasting delayed FL and UC transients were measured with a time-gated iCCD camera New iStar DH340T (Andor) after exciting the samples with the emission of a tunable-wavelength optical amplifier (Ekspla) pumped by a nanosecond Nd^3+^:YAG laser (wavelength – 640 nm, pulse duration – 5 ns, repetition rate – 1 kHz). FL and UC quantum yields were determined by utilizing an integrating sphere (Sphere Optics) coupled with the CCD spectrometer PMA-12 *via* an optical fiber and carrying out the procedure described by Mello *et al.*^53^ The UC quantum yield is defined as the ratio of emitted UC photons to total absorbed photons. Thus, the UC yield can utmost reach 50%.

### DFT calculations

Molecular geometry and singlet and triplet state energies of acene-based annihilators were modeled using the quantum chemistry program ORCA.^54^ DFT geometry optimization for each molecule was performed at the B3LYP/6-31G(d)^43–45^ level in a vacuum. Later, time-dependent density functional theory (TD-DFT) calculations were performed to extract singlet and triplet state energies.

## Author contributions

LN, ER, PB, KMP, and KK conceptualized the idea of this work. ER and MD led the experimental work. LN and ER performed DFT calculations. LN, PB, and KMP wrote the first draft of the manuscript and revised it after consulting with all authors. All authors contributed to the analysis of the results.

## Conflicts of interest

There are no conflicts to declare.

## Supplementary Material

TC-011-D3TC03158F-s001
