# Supplementary material for: The statistical probability factor in triplet mediated photon upconversion: a case study with perylene
Source: J Mater Chem C Mater. 2023 Oct 2;11(42):14826–32. doi: 10.1039/d3tc03158f (PMC10621484; doi:10.1039/d3tc03158f)
Supplement: TC-011-D3TC03158F-s001 [file TC-011-D3TC03158F-s001.pdf]

Electronic Supplementary Material (ESI) for Journal of Materials Chemistry C.
This journal is © The Royal Society of Chemistry 2023

Supporting information

Statistical Probability Factor in Triplet Mediated Photon Upconversion: A Case Study with Perylene

Lukas Naimovičius,[ab] Edvinas Radiunas,[b] Manvydas Dapkevičius,[b] Pankaj Bharmoria,[a] Kasper Moth-Poulsen*[acde] and Karolis Kazlauskas*[b]

[a] Institute of Materials Science of Barcelona, ICMAB-CSIC, Bellaterra, Barcelona, 08193, Spain.
[b] Institute of Photonics and Nanotechnology, Vilnius University, Saulėtekio av. 3, LT-10257 Vilnius, Lithuania.
[c] Catalan Institution for Research & Advanced Studies, ICREA, Pg. Llu´ıs Companys 23, Barcelona, Spain.
[d] Department of Chemical Engineering, Universitat Politècnica de Catalunya, EEBE, Eduard Maristany 10–14, 08019 Barcelona, Spain.
[e] Department of Chemistry and Chemical Engineering, Chalmers University of Technology, Kemivagen 4, Gothenburg 412 96, Sweden

*E-mails – kmothpoulsen@icmab.es; karolis.kazlauskas@ff.vu.lt

Contents

Upconversion and Phosphorescence 2
1. UC Emission and PdTPBP Phosphorescence Spectra 2
2. Relative UC Quantum Yield Measurement 2
3. Comparative UC and FL Spectra of Perylene 4
Excitation Beam Profiles 4
Statistical Probabilities of Acenes 5
Perylene-based TTA-UC Systems 5
Density Functional Theory Calculations 6
Error Estimation for Statistical Probability 7

Upconversion and Phosphorescence

1. UC Emission and PdTPBP Phosphorescence Spectra

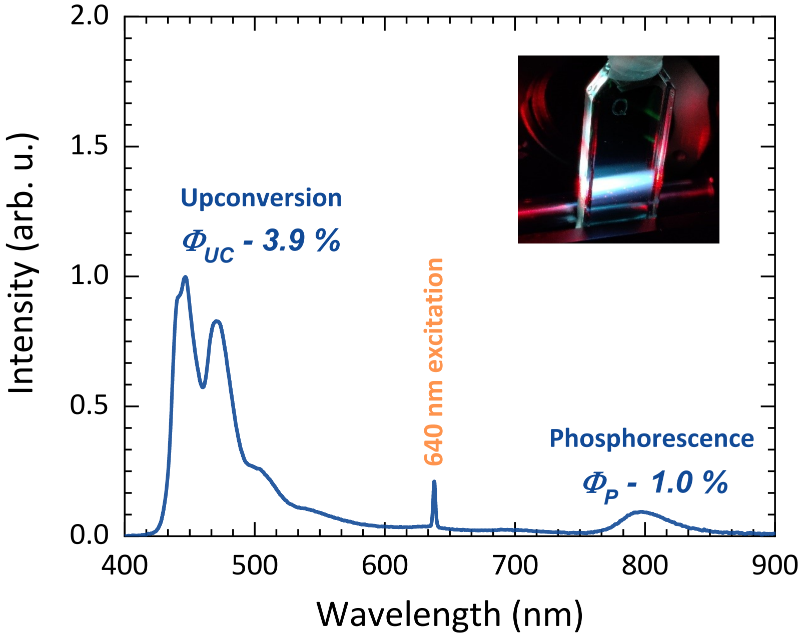

Fig. S1. UC emission spectrum (uncorrected for reabsorption) of the studied perylene:PdTPBP solution in THF at 1×10^{-4} M and 1×10^{-5} M concentrations, respectively. (λ_{ex} = 640 nm CW laser).

2. Relative UC Quantum Yield Measurement

we employed the same relative method as used in the study by Monguzzi et al. to estimate Φ_{UC}, to confirm our conclusions. For this experiment, we selected the Nile Blue as the reference with Φ_{FL} = 27%[1] in 0.1 M HCl in ethanol, considering its similar absorption to that of PdTPBP (Fig. S1a). The UC sample and the reference were excited using a 638 nm laser (Fig. S1a) under identical configuration and excitation intensity.

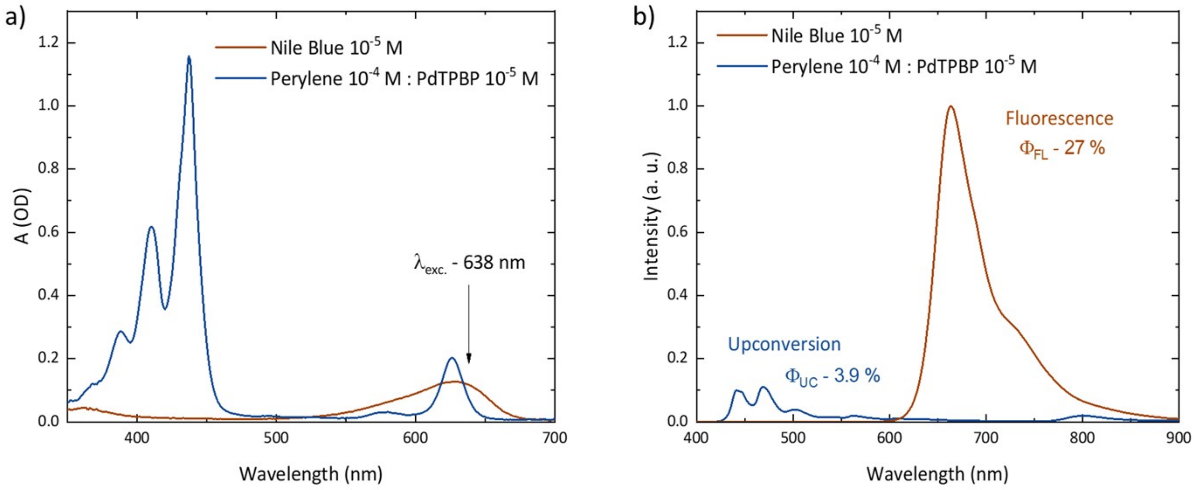

Fig. S2 a) Absorption spectra of Nile Blue at 10^{-5} M in 0.1 M HCl in ethanol and Perylene:PdTPBP UC solution in THF at 10^{-4} M and 10^{-5} M, respectively. b) Photoluminescence spectra of upconversion sample (blue line), and fluorescence spectrum of reference, nile blue dye (brown line) under 638 nm laser excitation. ϕ_{UC} and ϕ_{FL} indicated.

After recording the upconversion and reference's fluorescence spectra (Fig. S2b), the relative Φ_{UC} was calculated using eq. 1 and the parameters in Table R1.

$$\Phi_S = \Phi_R \left(\frac{I_S}{I_R}\right)\left(\frac{1 - 10^{-A(R)}}{1 - 10^{-A(S)}}\right)\left(\frac{n_S}{n_R}\right)^2 \tag{1}$$

Here Φ_S and Φ_R are quantum yields of the sample and reference (Nile Blue standard), I_S and I_R – spectrally integrated intensities of the sample and reference, A_S and A_R – absorbance of the sample and reference, n_S and n_R – refractive indices of the sample and reference solutions (in this case, indices of THF and ethanol were used).

Table S1 Main parameters (absorption, integrated intensity, Φ_{FL}, and refractive index) of the relative quantum yield measurement used to determine the Φ_{UC}.

Measurement	Sample	A (OD)	Normalized Integ. Int.	Φ_{FL} (%)	n	Φ_{UC} (%)
1	Nile Blue	0.118	1	27	1.3614	
	Perylene:PdTPBP	0.0759	0.091		1.4072	**3.90**
2	Nile Blue	0.118	1	27	1.3614	
	Perylene:PdTPBP	0.0759	0.093		1.4072	**4.00**
3	Nile Blue	0.118	1	27	1.3614	
	Perylene:PdTPBP	0.0759	0.089		1.4072	**3.83**

The relative quantum yield measurement was performed three times, resulting in an average Φ_{UC} value of ~3.9 ± 0.3%. This is similar to the result obtained using the absolute method

(3.9 ± 0.2%). Thus, we conclude that the Φ_{UC} results are consistent when employing both measurement methods.

3. Comparative UC and FL Spectra of Perylene

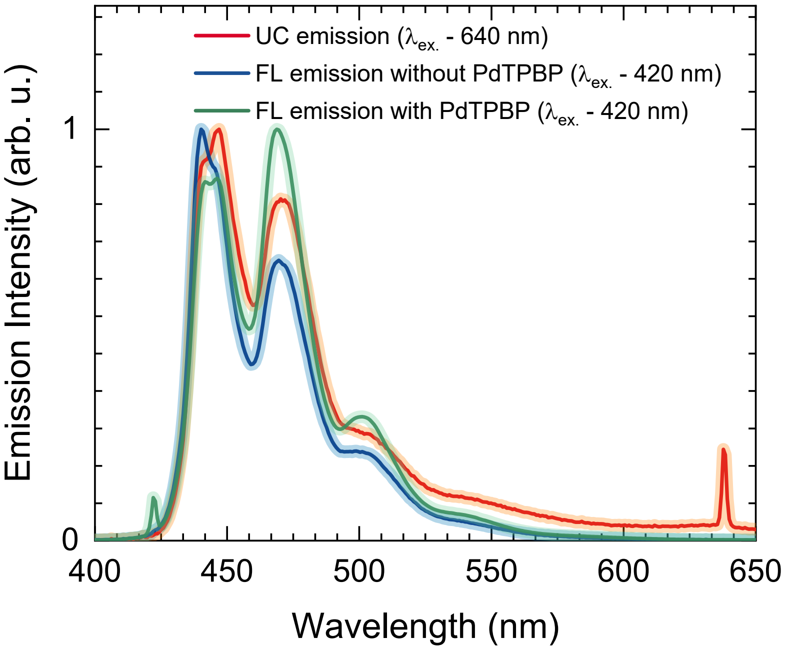

Fig. S3. Comparative UC emission and fluorescence spectra of perylene in THF at 1 $\times$ 10^{-4} M.

Excitation Beam Profiles

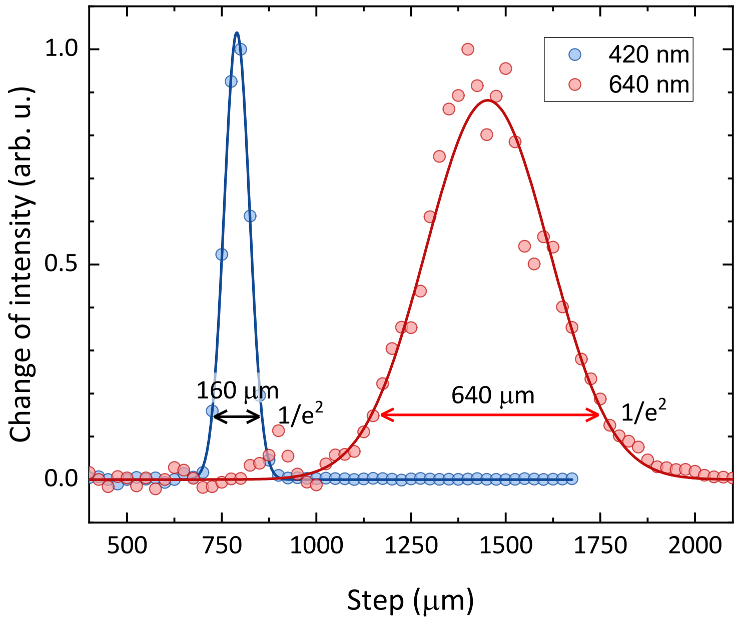

Fig. S4. Beam profiles of 420 nm and 640 nm CW laser excitation. Beam profiles fitted with Gaussian distribution. Beam diameters for each excitation wavelength are indicated as $1/e^2$.

Statistical Probabilities of Acenes

Table S2 Reported statistical probability (f) values of acene-based annihilator chromophores. Asterisk (*) marks the values determined by us from available data.

TIPS-naph	DPA	Perylene	BPEA	Rub	tb-Rub
54 %[3]	28 %[4]	16 %[5]*	5 %[4]	15.5 %[6]	5.3 %[6]
	34 %[4]	22 %[5]*	5.6 %[4]		
	48 %[7]*	25 %[5]*	8.6 %[8]*		
	52 %[9]	80 %[1]			
		100 %[1]			

Table S3 Example of statistical probability estimation from the available data in ref. 8.

Solvent	ϕ_{ISC}	ϕ_{TET}	ϕ_{FL}	$I_{th(reported)}$	$I_{th(reassessed)}$	$\phi_{UC(reported)}$	ϕ_{UC}^{∞}	ϕ_{TTA}	f
Toluene	100%	100%	85%	0.26 W/cm^2	0.35 W/cm^2	4.2%	6.9%	100%	**16%**

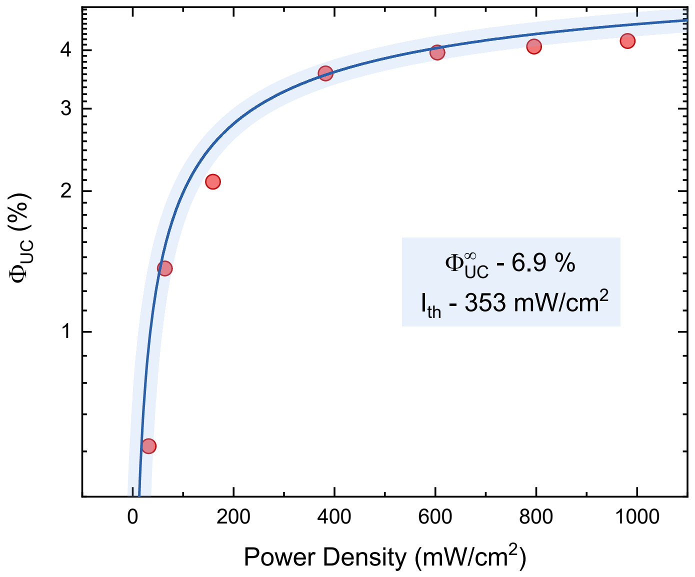

Fig. S5. ϕ_{UC} dependence on I_{ex} of the perylene:I_2-Bodipy UC system available elsewhere[5]. Data fitted according to equation 6 is represented as the blue line. UC threshold (I_{th}) and maximum UC quantum yield (ϕ_{UC}^{∞}) are indicated.

Perylene-based TTA-UC Systems

Table S4 Reported UC quantum yield (ϕ_{UC}), threshold (I_{th}) and statistical probability (f) values of perylene-based TTA-UC systems. Asterisk (*) marks the values determined by us from available data.

Publication	ϕ_{UC} (%)	I_{th} (W cm^{-2})	f (%)
Zhang et al.[5]	2.8 - 4.3	0.26	16 - 25*
Börjesson et al.[10]	9	5.0*	-
Börjesson et al.[11]	3.2 - 5.0	0.02	-
Monguzzi and Duan et al.[12]	42	1.3	-
Börjesson et al.[13]	2.0 - 6.0	0.02	-
Monguzzi et al.[1]	38	-	80 - 100
Baluschev et al.[14]	4.9	-	-
Han et al.[15]	3.5	0.081	-
Kim et al.[16]	3.1	-	-

This work	**4.1 ± 0.2**	**1.0**	**17.9 ± 2.1**

Density Functional Theory Calculations

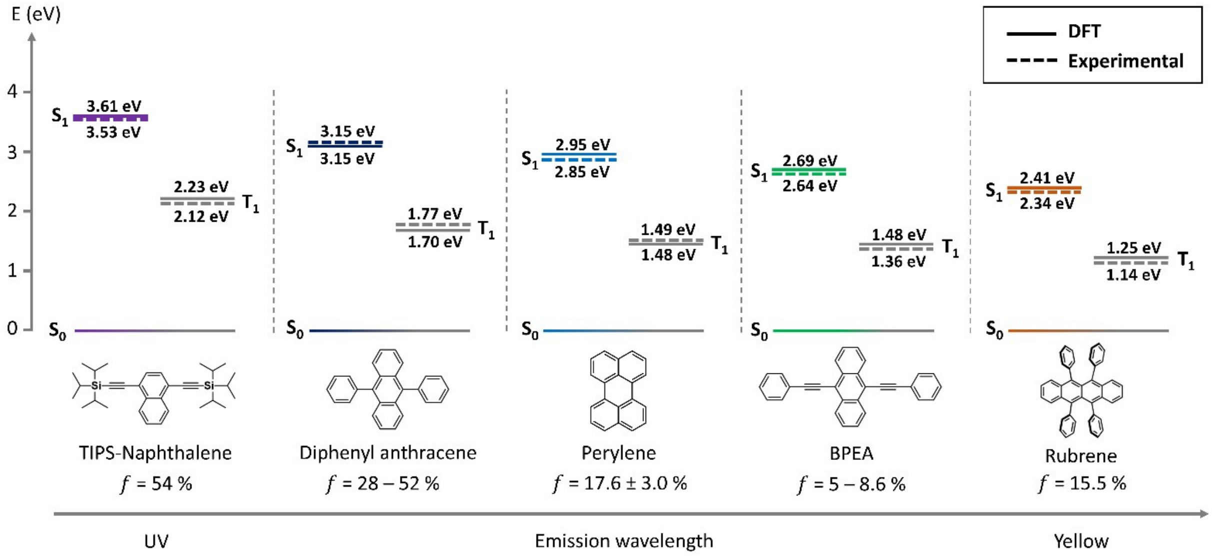

Fig. S6. Comparison of calculated and experimental S_1 and T_1 energy states of different acene-based annihilator chromophores emitting from UV to yellow region of spectrum. a) TIPS-Naphthalene; b) Diphenyl anthracene; c) Perylene; d) BPEA; e) Rubrene.

Table S5 Comparison of calculated and experimental S_1 and T_1 energy states of different acene-based annihilator chromophores emitting from UV to yellow region of spectrum.

Annihilator	T_1 (exp.)	T_1 (calc.)	S_1 (exp.)	S_1 (calc.)
TIPS-Naphthalene	2.12[17]	2.23	3.53[17]	3.61
Diphenyl anthracene	1.77[18]	1.70	3.15[7]	3.15
Perylene	1.49[19]	1.48	2.85	2.95
BPEA	1.36-1.82[20]	1.48	2.64[21]	2.69
Rubrene	1.14[22,23]	1.25	2.34[24]	2.41

Error Estimation for Statistical Probability

The error of the statistical probability factor was evaluated *via* this equation:

$$\Delta f = f\sqrt{\left(\frac{\Delta\phi_{UC}}{\phi_{UC}}\right)^2 + \left(\frac{\Delta\phi_{TET}}{\phi_{TET}}\right)^2 + \left(\frac{\Delta\phi_{TTA}}{\phi_{TTA}}\right)^2 + \left(\frac{\Delta\phi_{FL}}{\phi_{FL}}\right)^2}$$

References

1 S. Hoseinkhani, R. Tubino, F. Meinardi and A. Monguzzi, *Phys. Chem. Chem. Phys.*, 2015, **17**, 4020–4024.

2 R. Sens and K. H. Drexhage, *J. Lumin.*, 1981, **24**–**25**, 709–712.

3 A. Olesund, J. Johnsson, F. Edhborg, S. Ghasemi, K. Moth-Poulsen and B. Albinsson, *J. Am. Chem. Soc.*, 2022, **144**, 3706–3716.

4 V. Gray, A. Dreos, P. Erhart, B. Albinsson, K. Moth-poulsen and M. Abrahamsson, 2017, 10931–10939.

5 Q. Zhou, M. Zhou, Y. Wei, X. Zhou, S. Liu, S. Zhang and B. Zhang, *Phys. Chem. Chem. Phys.*, 2017, **19**, 1516–1525.

6 E. Radiunas, S. Raišys, S. Juršenas, A. Jozeliunaite, T. Javorskis, U. Šinkeviciute, E. Orentas and K. Kazlauskas, *J. Mater. Chem. C*, 2020, **8**, 5525–5534.

7 A. Olesund, V. Gray, J. Mårtensson and B. Albinsson, *J. Am. Chem. Soc.*, 2021, **143**, 5745–5754.

8 D. Beljonne and A. Rao, *ACS Mater. Lett.*, 2019, **1**, 660–664.

9 A. Monguzzi, R. Tubino, S. Hoseinkhani, M. Campione and F. Meinardi, *Phys. Chem. Chem. Phys.*, 2012, **14**, 4322–4332.

10 C. Ye, V. Gray, K. Kushwaha, S. Kumar Singh, P. Erhart and K. Börjesson, *Phys. Chem. Chem. Phys.*, 2020, **22**, 1715–1720.

11 A. J. Carrod, A. Cravcenco, C. Ye and K. Börjesson, *J. Mater. Chem. C*, 2022, **10**, 4923–4928.

12 W. Sun, A. Ronchi, T. Zhao, J. Han, A. Monguzzi and P. Duan, *J. Mater. Chem. C*, 2021, **9**, 14201–14208.

13 C. Ye, V. Gray, J. Mårtensson and K. Börjesson, *J. Am. Chem. Soc.*, 2019, **141**, 9578–9584.

14 A. Vasilev, R. Dimitrova, M. Kandinska, K. Landfester and S. Baluschev, *J. Mater. Chem. C*, 2021, **9**, 7119–7126.

15 L. Zeng, L. Huang, W. Lin, L. H. Jiang and G. Han, *Nat. Commun.*, 2023, **14**, 1102.

16 O. S. Kwon, J. H. Kim, J. K. Cho and J. H. Kim, *ACS Appl. Mater. Interfaces*, 2015, **7**, 318–325.

17 N. Harada, Y. Sasaki, M. Hosoyamada, N. Kimizuka and N. Yanai, *Angew. Chemie - Int. Ed.*, 2021, **60**, 142–147.

18 S. K. Chattopadhyay, C. V. Kumar and P. K. Das, *Chem. Phys. Lett.*, 1983, **98**, 250–254.

19 Y. Sasaki, M. Oshikawa, P. Bharmoria, H. Kouno, A. Hayashi-Takagi, M. Sato, I. Ajioka, N. Yanai and N. Kimizuka, *Angew. Chemie - Int. Ed.*, 2019, **58**, 17827–17833.

20 T.-S. Fang, J. Lin, R. Schneider, T. Yamada and L. A. Singer, *Chem. Phys. Lett.*, 1982, **92**, 283–287.

21 V. Gray, A. Dreos, P. Erhart, B. Albinsson, K. Moth-poulsen and M. Abrahamsson, *Phys. Chem. Chem. Phys.*, 2017, **19**, 10931–10939.

22 W. G. Herkstroeter and P. B. Merkel, *J. Photochem.*, 1981, **16**, 331–341.

23 D. K. K. Liu and L. R. Faulkner, *J. Am. Chem. Soc.*, 1977, **99**, 4594–4599.

24 E. Radiunas, M. Dapkevičius, S. Raišys, S. Juršenas, A. Jozeliunaite, T. Javorskis, U.

Šinkevičiute, E. Orentas and K. Kazlauskas, *Phys. Chem. Chem. Phys.*, 2020, **22**, 7392–7403.
